# Effect of Educational Program, Based on PRECEDE and Trans-Theoretical Models, on Preventing Decline in Regular Physical Activity and Improving it among Students

**Published:** 2017-04-10

**Authors:** Masomeh Rostami-Moez, Forouzan Rezapur-Shahkolai, Seyyed Mohammad Mahdi Hazavehei, Manoochehr Karami, Akram Karimi-Shahanjarini, Farzad Nazem

**Affiliations:** ^1^ Department of Public Health, School of Public Health, Hamadan University of Medical Sciences, Hamadan, Iran; ^2^ Research Center for Health Sciences, Hamadan University of Medical Sciences, Hamadan, Iran; ^3^ Social Determinants of Health Research Center, Hamadan University of Medical Sciences, Hamadan, Iran; ^4^ Department of Epidemiology, School of Public Health, Hamadan University of Medical Sciences, Hamadan, Iran; ^5^ Department of Sport Physiology, Section of Health Science, Faculty of Physical Education and Sport Sciences, Bu-Ali Sina University, Hamadan, Iran

**Keywords:** Adolescents, Female, Health Promotion, Randomized Controlled Trial, Iran

## Abstract

**Background:** Adolescents especially middle schoolgirls do not follow 60 min of regular physical activity (RPA), recommended by WHO (World Health Organization), and endure physical activity decline. Using theory-based interventional program, considering the age of decline in RPA, seems to be effective. The aim of this study was to determine the effects of educational program, based on PRECEDE and Trans-Theoretical models, on preventing decline in RPA and improving it among 7th grade girl students.

**Study design:** Randomized controlled trial.

**Methods:** This study was conducted on 7^th^ grade girl students in Hamadan, west of Iran from 2015- 16. Participants were divided into intervention (N=179) and control (N=165) groups by random assignment. Physical activity questionnaire for adolescents and the model-based questionnaire were used before and after intervention. Intervention included two months education and six months follow up. The effects of intervention were determined by statistical test and analysis of covariance using SPSS version 16.

**Results:** The higher change in the mean scores was observed in self-efficacy (0.86), counter condition (0.66) and helping relationship (0.57) in the intervention group (*P*≤0.001). In addition, a significant difference (*P* ≤ 0.01) was observed between the two groups 6 months after the intervention in all constructs of model. The mean score of doing physical activity in the intervention group increased from 2.50 to 3.17 that it was differed significantly from the control group (*P*≤0.001).

**Conclusions:** School based intervention using PRECEDE and Trans-Theoretical models might prevent girl students’ RPA decline and improve their RPA.

## Introduction

The importance of regular physical activity (RPA) on physical and mental health is documented. Inadequate levels of RPA among adolescents is a global concern^1^ and can lead to many non-communicable diseases, such as metabolic syndrome, cardiovascular disease and obesity, in adulthood^2-4^. Although World Health Organization (WHO) recommended at least 60 min of moderate to vigorous physical activity (MVPA), leading to increase heart rate and breathing for young people^5^, but 80% of them do not obey it^1^, and only 8% of American adolescents do this^6^. Iranian teenagers, especially girls’ RPA is lower than this value^7^. Researches also reported a drop in RPA during adolescence^8^, which is more common in girls^1, 8^.

This study is part of a research project, and the diagnostic evaluation done already showed the effectiveness of predisposing, enabling, and reinforcing factors, using in PRECEDE model, in performing RPA in adolescents. Furthermore, a cross-sectional study, as a part of the diagnostic evaluation, showed that only 6.3% of girls at the age of 10 to 16 yr perform one hour per day MVPA and the deep decline in physical activity (PA) was seen in eighth grade ^9^. Using multiple models for promoting PA has higher success ^10-12^.

PRECEDE model, can provide the proper structure to formulate appropriate strategies in developing and evaluating health promotion interventions in adolescents. This model includes constructions of social assessment (quality of life), epidemiological assessment (body mass index review is here), environmental assessment (such as schools conditions) and behavioral assessment (such as RPA), ecological and educational assessment (predisposing, reinforcing and enabling factors), process, impact and outcome evaluation,^14^. PRECEDE model was used in different health promotion interventions^15-19^.

**Figure 1:**
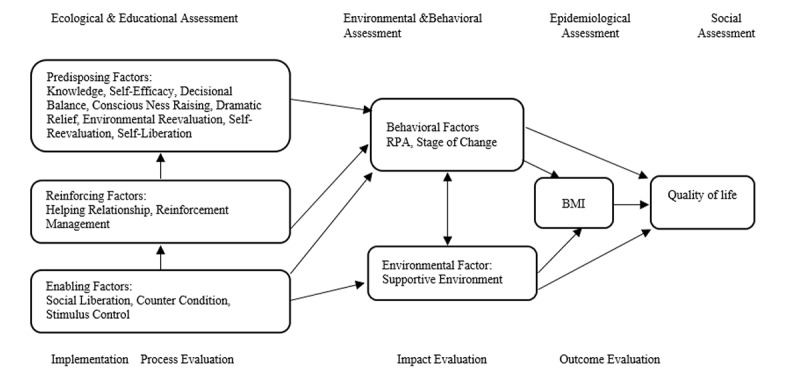
Intervention model for preventing regular physical activity decline among 7^th^ grade female students, designed in PRECEDE Framework combining with constructs of Trans-Theoretical Model

Trans-Theoretical Model (TTM), as a useful model while there is resistance to behavior change, suggests that persons for their behavioral changes pass through five stages: pre-contemplation, contemplation, preparation, action and maintenance^13, 20^. Other components of this model include the decisional balance (benefits and obstacles of a behavior), self-efficacy (confidence in the ability to perform a behavior) and process of change (including 5 behavioral and 5 cognitive process) that individuals use during passing the pre-contemplation stage to maintenance^21^. Cognitive processes include consciousness raising, dramatic relief, self-reevaluation and environment reevaluation, self-liberation. Behavioral processes include helping relationships, counter condition, reinforcing management, stimulus control and social liberation,^20^. Parental support or rewards are examples of behavioral processes. This model has been used in studies of PA ^22, 23^. Therefore, combining the above-mentioned two models can offer a good design for an intervention program (Figure 1).

In this study PRECEDE model was used for planning the intervention and TTM for educational assessment. Besides, to research team knowledge, in most previous studies, emphasis was on promoting RPA, while in this study preventing the age-related decline of RPA was considered as well.

This study aimed to determine the effect of educational program based on combining PRECEDE model and TTM for preventing PA decline in 7th grade girl students.

## Methods

### Participants

This randomized controlled trial was performed among 314 female students in 7^th^ grade girl students in the city of Hamadan, west of Iran from October 2015 to May 2016. As diagnostic evaluation showed the deep decline in RPA in the 8^th^ grade among female students^9^, to prevent it, the effective interventions needed to be implemented in seventh grade. The program implemented based on combining PRECEDE and TTM for 8 months.

Students participated in this study voluntarily. Informed consents were obtained before the study from both students and their parents or guardians. The study was approved by Ethics Committee of University of Hamadan Medical Sciences (number= 16-35-9-5301). In addition, this study is registered in Iran clinical trials registry (code=2015042921155N2).

To select the intervention and control groups, first, the two regions of Hamadan Education Department were divided randomly into intervention and control areas in order to prevent the dissemination of information. Second, in each region schools were divided into three categories (good, intermediate and inappropriate sport related facilities and places) using data from the Department of Education. Third, from each category, one school was selected randomly (3 schools as intervention and 3 schools as control groups) and from each school, two classes of seventh grade were randomly selected to participate in the program. The required sample size was 135 students for each group. On the other hand, in each school, 45 students were required, students’ number in classes was 25 to 33, and the intervention was not possible to be conducted outside of school hours. Therefore, two classes from each school were selected randomly. Thus, the sample size increased to 179 students for intervention group and 165 students for control group. A number of 15 students in the intervention group and 15 students in the control group were excluded based on inclusion and exclusion criteria. Finally, 150 students in the control group and 164 students in the intervention group participated in the study.

**Figure 2:**
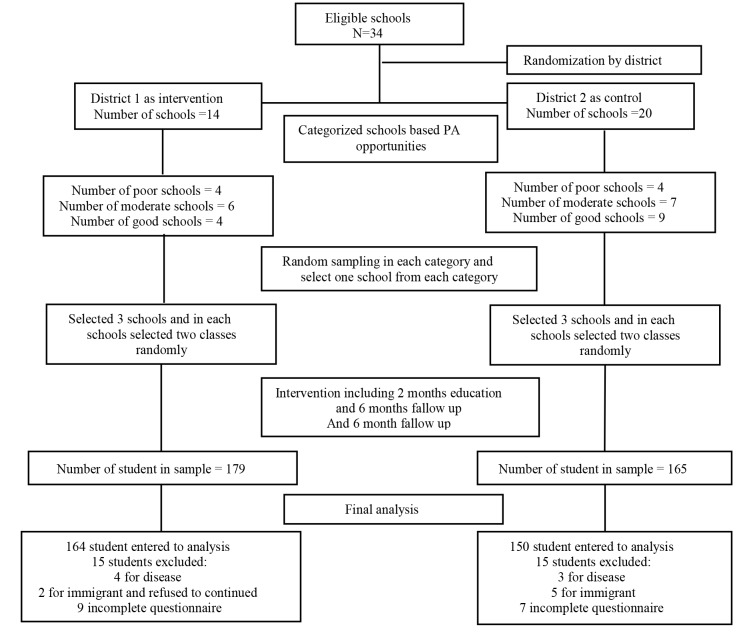
Flow chart of sampling procedure for the interventional study

Inclusion criteria included seventh grade school girls in Hamadan, volunteered to participate in the study, the agreement of the parents and having no injury or illness that prevents PA, respectively. Exclusion criteria included not attending in the class and intervention programs, did not complete the questionnaire and migration. Figure 2 shows the flow chart of sampling procedure in this interventional study.

### Measures

Three questionnaires were used to collect data as follows:

A. Demographic questionnaire, containing questions in terms of parents’ jobs, parents' educational level, pubertal status, membership in a sports club and household income.
B. The PA questionnaire for adolescent (PAQ_A) was used to assess PA^24^. PAQ_A is a‏ self-reported about previous week PA recall. It contains nine questions starting with a checklist of last week sports (“no”, “1-2”, “3-4”, “5-6”, “7 or more times”), questions 2-7 asking about how often adolescents were active during physical education class, lunchtime, after school, evenings, weekend, describe their best and how often they were active in each day of their last week. Each question of PAQ_A has five choices in 5-point Likert scale (e.g., “never”, “hardly ever”, “sometimes”, quite often “and “always”). The final question requests if anything banned the adolescents to perform their usual PAs (“yes”, “no”). Total score is calculated as mean score of questions in which higher scores indicate higher PA levels. Previous studies have shown internal consistency (α = 0.79 to 0.89)^25^, and validity (rho= 0.47 for total PA and rho = 0.49 for MVPA) with spearman correlation between PAQ-A and accelerometer ^26^.
C. TTM based questionnaires contain 45 questions used for educational assessment of PRECEDE model ^21, 27, 28^. Stage of change (SOC) asks with 5 statements that students select one of the stages describing their present MVPA as an hour a day so that heart rate goes up and breathing becomes a little difficult as follows: I performed RPA more than 6 months (maintenance), I performed RPA less than 6 months (action), I intend to perform RPA within next month (preparation), I intend to perform RPA within 6 months (contemplation), I do not intend to engage in RPA within 6 months (pre-contemplation)^21^.

Self-efficacy measured by 6 questions in 5-point Likert scale ranging from completely confident (5 points) to not at all confident (1 point), how confident you are to perform RPA in certain circumstances for example when you spend time with your friends^21^, decisional balance is measured by 10 items (5 item for pros and five item for cons) in 5-point Likert scale ranging from extremely important to not important which means students defined the importance of each statement for decision making on exercising or not ^28^ and processes of change questionnaire include 10 process in 5-point Likert scale ranging from always (5 points) to never (one point)^27^. Processes of consciousness raising (3 questions), dramatic relief (3 questions), environmental re-evaluation (2 questions), self-reevaluation (3 questions) and self-liberation (3 questions) were questioned as predisposing factors. Processes of stimulus control (2 questions), counter condition (3 questions) and social liberation (3 questions) as enabling factors and processes of reinforcing management (3 questions) and helping relationships (2 questions) as reinforcing factors were questioned. Also, based on literature review 11 questions were produced by researchers to assess knowledge about PA. Each correct answer scored one and wrong answer scored zero. Face validity, content validity with 10 expert (CVI & CVR˃0.80) and confirmatory factor analysis (χ2= 1726.35, df =850 χ2/df=2.03, RMSEA=0.000) had been assessed in previous cross-sectional study in diagnostic evaluation^9^. Reliability was assessed by internal consistency (α=0.71) on 30 students who were non-participants in this study.

Students’ height and weight were measured by a digital scale and a stadiometer before and at the end of the study by the first researcher. Body Mass Index (BMI) was calculated for each students (kg/m2) and BMI Z-score was considered from WHO growth chart data.

### Intervention program

As the educational assessment phase of PRECEDE model, revealed to be effective predisposing, enabling, and reinforcing factors in performing RPA in adolescents, the training program based on PRECEDE and TTM was planned and implemented in this study. The program included 1 hour training per week for 2 months (8 hours, totally) in the field of physical activity pyramid, the importance and benefits of adequate PA, issues and problems of inactivity and insufficient PA, how to do proper exercise and daily PA schedule. Methods used were lecture, group discussion, poster (physical activity pyramid) and pamphlets (the importance, benefits and how to perform aerobic activity, stretching and strengthening), a daily log book for documenting PA, practical training sessions for aerobic activities, stretching and strengthening muscles based on the objectives of the program by a trained coach in school (predisposing and enabling factors). Friendship teams were formed to walk to school. Exhibition of PA (wall newspapers and‏ handcrafts) was held. Workout CD and sport oriented music was used (reinforcing and enabling factors) in the 8^th^ educational session. After that, the reminder bracelets with the sports sign and the word "be active” were distributed among students as a reminder and commitment improver for RPA (self-liberation).‏ In addition, the logbooks of PA were checked by principal researcher, each week for about 6 months, and students’ problems were solved and they were encouraged during this period.

Separately, for teachers, school staffs and students’ parents as reinforcing agents who affect students’ living environment (create supportive environments); two educating sessions were held. Educational content included importance of teenagers RPA and supporting them, duration and the way of performing RPA. In addition, special pamphlet for parents was sent to them through students and all pamphlets for staffs studying were placed in the school office. PA related banner with content of 60 min moderate to vigorous daily PA was installed as a reminder and for changing the environment to persuade students to do RPA in the hallway of the schools in intervention group. The students were followed for 6 months and then both groups completed questionnaires again. Regarding ethical considerations, after the completion of the study, the control group received educational materials and one session of education.

### Data analysis

The data of 314 students were analyzed finally (164 in intervention and 150 in control group). Statistical analyses were performed with IBM‏ statistic, SPSS version 20 (SPSS Inc., Chicago, IL, USA). Comparability of baseline characteristics of study groups was assessed using chi-square. Analysis of Covariance (ANCOVA) was used for comparison between-group differences on outcomes (PA, BMI and constructs of TTM in educational diagnosis of PRECEDE model). We first examined if the intervention and the control group differed at baseline on any of the study variables. Chi square test was used for differences between the stages of change. In all analyses, the level of significance was set at *P*< 0.05.

## Results

All of students had about 13 years old. Demographic details of participants‏ are presented in Table 1. There were no significant differences between the two groups before intervention in terms of demographic characteristics. Additionally, at baseline, there were no differences in PA and BMI between two groups.

**Table 1:** Distribution of frequency and percentage of basic variables in 7^th^ grade female students in Hamadan

**Variables**	**Intervention**		**Control**		***P*** ** value**
	**Number**	**Percent**	**Number**	**Percent**	
Mother education					0.341
Up to diploma	84	54.5	87	60.0	
‏≤12 yr	70	45.5	58	40.0	
Father education					0.863
Up to diploma	83	54.2	79	55.2	
‏≤12 yr	70	45.8	64	44.8	
Father job					0.370
Self-employment	89	55.6	88	61.1	
Employment	42	26.3	28	19.4	
Worker	29	18.1	28	19.4	
Mother job					0.342
Employment	27	16.5	19	12.7	
Unemployment	137	83.5	131	87.3	
Puberty statue					0.604
Thelarche	4	2.5	6	4.0	
Pubarche	44	27.2	45	30.0	
Menarche	114	70.4	99	66.0	
Being member of sports team					0.114
Yes	32	19.8	41	27.3	
No	130	80.2	109	72.7	
Family economic statue					0.229
low	21	14.4	10	7.9	
Moderate	61	41.8	64	50.8	
Good	55	37.7	42	33.3	
Excellent	9	6.2	10	7.9	

Considering educational program, Table 2 shows the details of mean score and standard deviation and significant levels in predisposing, enabling, and reinforcing factors by TTM constructs in both groups. After educational intervention, ANCOVA analysis revealed that mean scores in the intervention group were significantly different from those in the control group in all constructs except cons. BMI in intervention group had no differences after intervention but in control group it raised and BMI increase was significant (*P*-value=0.002). RPA significantly increased in intervention group and therefore, the mean score differences between two groups were significant (*P*-value<0.001).

**Table 2:** Predisposing, Reinforcing and Enabling Factors, Physical Activity and Body Mass Index among the students before and after the Intervention in the Intervention and Control Groups

**Variables**	**Intervention**				**Control**				***P*** ** value**
	**Before**		**After**		**Before**		**After**		
	**Mean**	**SD**	**Mean**	**SD**	**Mean**	**SD**	**Mean**	**SD**	
Predisposing factors									
Pros	4.14	0.76	4.43	0.60	4.26	0.73	4.23	0.81	0.001
Cons	2.90	0.77	2.74	0.94	2.84	0.85	2.78	0.82	0.336
Self-efficacy	3.04	0.88	3.90	0.84	3.09	0.92	3.01	0.90	0.001
Consciousness raising	3.54	1.02	3.98	0.97	3.82	1.03	3.79	1.01	0.002
Dramatic relief	3.82	1.07	4.09	0.93	3.60	1.16	3.65	1.10	0.001
Environment reevaluation	3.87	1.16	4.22	0.88	3.81	1.14	3.95	1.15	0.001
Self-reevaluation	4.47	0.79	4.42	0.80	4.48	0.82	4.22	0.95	0.010
Self-liberation	3.95	0.92	4.20	0.85	4.21	1.02	3.95	0.88	0.001
Knowledge	4.33	1.90	5.60	1.60	4.59	2.11	4.48	2.17	0.001
Reinforcing factors									
Helping relationship	3.25	1.28	3.82	1.15	3.62	1.32	3.40	1.33	0.001
Reinforcing management	4.25	0.84	4.35	0.79	4.23	0.89	4.12	0.99	0.003
Enabling factors									
Counter condition	3.15	1.14	3.81	1.03	3.35	1.11	3.29	1.21	0.001
Social liberation	3.94	0.86	4.19	0.77	4.08	0.89	3.96	1.01	0.022
Stimulus control	4.17	1.13	4.20	1.01	4.05	1.22	3.84	1.35	0.025
Behavioral factor									
Physical activity	2.50	0.60	3.17	0.64	2.48	0.60	2.49	0.69	0.001
Epidemiologic diagnosis									
Body mass index	19.02	4.15	19.14	4.06	19.97	3.88	20.53	3.93	0.002

Before the intervention, there were no differences between two groups in SOC. Distribution of SOC before and after intervention showed that indicates shifted to higher level for performing 60 min moderate to vigorous physical activity (MVPA) in intervention group (Table 3). Most of students were at action and maintenance stages (N=97; 59.2%). Before the intervention, 26 students (15.9%) were at the pre-contemplation and contemplation stages but after intervention, they decreased to 13 students (7.9%). Inversely in control group, students who were at the pre-contemplation and contemplation staged 23 students (22.4%) before the intervention increased to 47 students (31.3%) after the intervention.

**Table 3:** Stage of change and physical activity mean score before and after the intervention between intervention and control groups

**Stage of change**	**Intervention**		**Control**		***P*** ** value**
	**Before**	**After**	**Before**	**After**	
Pre action, n (%)	89 (54.30)	67 (40.80)	76 (51.70)	95 (63.30)	0.001
Action, n (%)	75 (45.70)	97 (59.20)	71 (48.30)	55 (36.70)	0.001
Pre-action, mean (SD) PA	2.37 (0.61)	3.09 (0.58)	2.25 (0.48)	2.31 (0.68)	0.001
Action, mean (SD) PA	2.65 (0.55)	3.26 (0.58)	2.72 (0.62)	2.68 (0.68)	0.001

Pre action (Pre-contemplation, Contemplation, Preparation); Action (Action, Maintenance); PA (physical activity); SD (standard deviation)

## Discussion

Before the study, the two groups in terms of demographic variables, model structures, mean score of PA and body mass index were not significantly different‏. The results of this study showed that the developing and implementing educational programs in accordance with PRECEDE model and TTM, produced a significant difference in the scores of predisposing, enabling and reinforcing factors and increased behavior of RPA among female students in the intervention group. In this study, after the intervention, scores of knowledge as a predisposing factor in the intervention group in comparison to the control group was increased. This result is consistent with previous studies ^29, 30^. Knowledge predicts PA ^31^. Respectively, self-efficacy as predisposing factor, helping relationships and strengthen management as reinforcing factors were increased. The influence of social support and self-efficacy has been shown in regular PA ^32^. Same as this study, previous study on 11-yr-old teenagers ^32^ suggests that social support from teachers (Reinforcing factors) and increased self-efficacy result in increasing PA (predisposing factors). Parents and teachers can be a role model and a source of social support for increasing PA in female adolescent. Raising awareness, verbal persuasion, goal setting, recording daily activities, skills training and doing PA, systematic strategies, were used to form and improve self-efficacy. Some studies have shown social support and self-efficacy mediate and predict PApromotion,^33, 34^. The counter condition and stimulus control for PA in the intervention group in comparison to the control group also were improved after the implementation of educational intervention (enabling factors). Stimulus control was a significant mediator for adolescents' PA ^35^. In this study, having a belief and commitment to PA (self-liberation) was significant. The difference between all processes of change for PA activity was significant after intervention. People use the processes of change for moving through SOC, such as self-liberation, reinforcement management, counter condition, stimulus control and helping relationship.

Set of interventions and changes based on combined model could change students’ behavior. Consequently, after the intervention, most students in the intervention group had reached the stage of action and maintenance and students in pre-contemplation, contemplation and preparation stages had been decreased. Contrary, students in the control group had been increased in pre-contemplation, contemplation and preparation stages and students in the maintenance stage had been decreased. In addition, a significant increase in mean score of PA score was observed. These results were consistent with Pops study ^22^. In the present study, not only decline was prevented in the intervention group but also PA increased. Results of the school-based intervention regarding their effect on rate and duration of PA were different in previous studies, as the interventions in some studies were effective and in some of them were not effective ^15^. Physical activity promotion can be attributed to environmental factors and social support in addition to individual factors. The school based interventions via access to the target group, the possibility of increasing hours of RPA or increasing the quality of physical education classes and providing relevant and diverse training sessions are effective strategies in increasing PA in adolescents. In addition to theoretical and practical training, improving the quality of physical education hours using trained coach besides to schools’ sport coach, keeping students active, encouraged and supporting them by researchers, teachers, parents and peers had a significant role to promote RPA ^16^.

In this study, BMI was not significantly increased in the intervention group, while significantly increased in the control group. It could be resulted from the impact of the intervention to increase RPA among participants in intervention group. As PRECEDE model argues about the problems of epidemiological and social resources, body mass index as an epidemiological problem wasinvestigated in this study. Johnson and colleagues showed TTM effectiveness in weight control via PA, nutrition and mental stress during their 24-month investigation and observed a significant difference between intervention and control groups in promoting PA and weight control^36^. Kattelmann and colleagues study, using PRECEDE model, showed increasing intensive activity among women in the intervention group comparing the control group^37^.

This study in spite of the strengths of preventing decline in RPA and improving it, based on strong models, had some limitations including conducting among girls and cannot being used for comparisons between both sexes. In addition, self-reported questionnaires may lead to some overestimates and using observational tools such as Actigraph or pedometer is recommended for future studies.

As the policy making and practical implications, regarding effectiveness of the school-based intervention in this study, using such a theory-based interventional program on RPA can be recommended among students in the community level and consideration of predisposing factors (such as self-efficacy), enabling factors (such as social liberation) and reinforcing factors (such as involving teachers, school staff and parents) can be helpful.

## Conclusions

Intervention, based on PRECEDE and TTM, not only could prevent decline in RPA, but also could improve it among studied female students. Although, the number of students who performed one hour of RPA (action and maintenance stages) increased in the intervention group of this study, but more programs are needed to achieve ideal goal (maintenance stage). The female students in seventh grade can follow recommended RPA for their health promotion.

## Acknowledgements

The authors would like to thank all of the participants and schools’ personnel.

## Conflict of interest statement

The authors declare there is no conflict of interest.

## Funding

This study is derived from the PhD thesis, approved and financially supported by the Vice-Chancellor of Research and Technology, Hamadan University of Medical Sciences study (number: 9308063798).

## Highlights

- Regular physical activity is a key factor in female adolescent health .
- Decline in physical activity occurs in adolescence period, which can prevent by appropriate intervention .
- PRECEDE and Trans-Theoretical models can provide suitable intervention framework to improve physical activity and prevent its decline in female students.
